# Crop Damage by Primates: Quantifying the Key Parameters of Crop-Raiding Events

**DOI:** 10.1371/journal.pone.0046636

**Published:** 2012-10-03

**Authors:** Graham E. Wallace, Catherine M. Hill

**Affiliations:** 1 Anthropology Centre for Conservation, Environment, and Development, Oxford Brookes University, Oxford, United Kingdom; 2 Division of Ecology and Evolution, Faculty of Natural Sciences, Imperial College London, Ascot, United Kingdom; University of North Carolina at Charlotte, United States of America

## Abstract

Human-wildlife conflict often arises from crop-raiding, and insights regarding which aspects of raiding events determine crop loss are essential when developing and evaluating deterrents. However, because accounts of crop-raiding behaviour are frequently indirect, these parameters are rarely quantified or explicitly linked to crop damage. Using systematic observations of the behaviour of non-human primates on farms in western Uganda, this research identifies number of individuals raiding and duration of raid as the primary parameters determining crop loss. Secondary factors include distance travelled onto farm, age composition of the raiding group, and whether raids are in series. Regression models accounted for greater proportions of variation in crop loss when increasingly crop and species specific. Parameter values varied across primate species, probably reflecting differences in raiding tactics or perceptions of risk, and thereby providing indices of how comfortable primates are on-farm. Median raiding-group sizes were markedly smaller than the typical sizes of social groups. The research suggests that key parameters of raiding events can be used to measure the behavioural impacts of deterrents to raiding. Furthermore, farmers will benefit most from methods that discourage raiding by multiple individuals, reduce the size of raiding groups, or decrease the amount of time primates are on-farm. This study demonstrates the importance of directly relating crop loss to the parameters of raiding events, using systematic observations of the behaviour of multiple primate species.

## Introduction

Understanding and addressing conflict between humans and wildlife due to crop-raiding is a crucial conservation issue [1], [2]. Crops near forest are often predictable and accessible sources of nutrition for wildlife [3], and extensive damage through raiding can adversely impact farmer livelihood [4], [5], compromise food security [6], reduce tolerance of wildlife [7], and undermine management strategies [8]. Conflict mitigation requires a comprehensive record of crop-raiding activity, including patterns of raiding, farmer and raider behaviour, crop losses, and the parameters of raiding events [9].

The literature on crop-raiding includes many accounts of non-human primates or other animals entering farms and raiding crops [10], [11], [12], [13]; however, these are typically indirect or anecdotal rather than systematic observations of behaviour. There is also little empirical analysis of which attributes of crop-raiding events (CREs) determine amount of crop loss. Although raider age and/or sex, group size, crop-raiding experience, and distance from forest potentially influence the extent of raiding at a farm [14], [15], [16], [17], [18], few studies quantify these or other parameters of CREs, or confirm links to the amount of damage that occurs during a CRE. This information is essential when developing techniques to protect crops because (i) deterrents can be designed to address specific raiding characteristics and (ii) methods reducing damage directly have the largest impact on yields and greatest value for farmers [19].

The effectiveness of crop-protection techniques is reflected in crop loss per unit of cost and farmer effort [9]. Therefore, quantifying the CRE parameters that determine damage to crops also measures deterrent efficacy. These parameters will be behavioural indices of the impact of deterrents and are likely to include how many individuals raid, how far they travel onto a farm, and how long they raid for. Related factors might include whether raids occur in series and/or age composition of the raiding group. Age probably correlates with raiding experience for primates consuming crops [3]; compared to novice raiders, primates with greater experience should access or process crop items more efficiently, and avoid detection by farmers more frequently or for longer durations. Parameter values may vary across species and/or circumstances, and collectively probably reflect the tactics used by raiding animals.

The research investigated the behaviour of multiple primate species to explore links between CRE characteristics and resultant damage to crops. The parameters of CREs that determine farmers’ losses were identified and quantified, to better understand which aspects of raider behaviour should be targeted by deterrents to reduce crop-raiding and manage conflict. This research is part of a study applying systematic observational methods to examine primate crop-raiding behaviour and develop effective conflict-mitigation techniques [9].

## Methods

### Study Site and Farms

**Figure 1:**
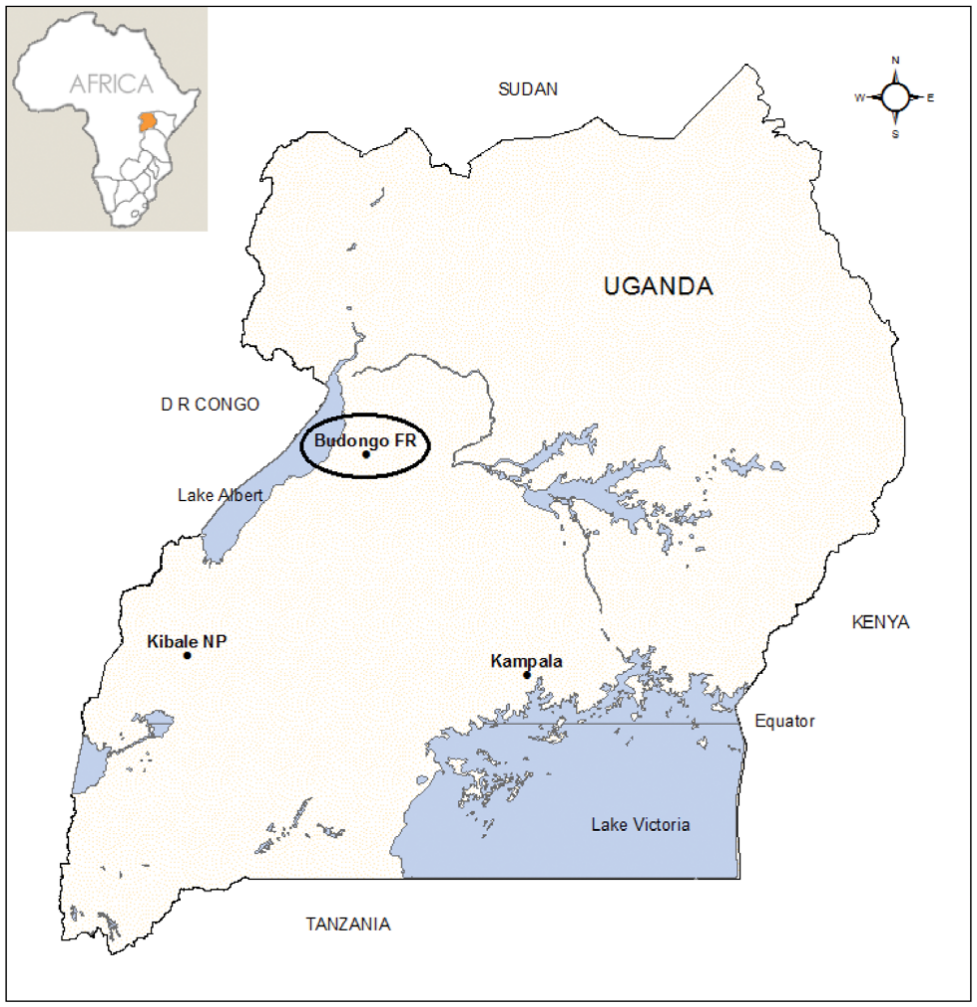
Map showing the location of Budongo Forest Reserve in western Uganda.

The research was conducted at forest-agriculture interfaces around Budongo Forest Reserve in the northern Albertine Rift, western Uganda (Figure 1). The reserve comprises almost 790 km^2^ of moist, semi-deciduous tropical forest and woodland managed for timber harvesting through selective logging since the 1920 s [20], [21]. Study farms were located across six villages within Nyabyeya parish (Table 1), which has an ethnically diverse human population reliant on artisanal farming [22]. Contingencies undermining crop yields directly impact local food security, and many farmers perceive crop-raiding by wildlife as the major threat to their livelihood [4]. Mean annual rainfall is 1,500 mm, peaking in April and October; mean monthly temperature is 21°C [23]. The primary crop-growing season is from March to September.

**Table 1:** Location of villages and dimensions of study farms.

Village	Latitude^1^	Longitude^1^	Study	Area	Perimeter	Forest edge^2^	Elevation^3^
			farms	(m^2^)	(m)	(m)	(m)
Nyakafunjo	1°41.741′N	31°32.451′E	Farm01	23,725	724	37	1,091
			Farm02	9,519	408	89	1,088
			Farm03	23,122	698	125	1,085
Marram	1°40.757′N	31°31.150′E	Farm04	28,302	823	146	1,087
			Farm05	36,396	1,036	93	1,088
Fundudolo	1°41.083′N	31°28.627′E	Farm07	26,843	726	196	1,055
			Farm08	1,629	190	116	1,045
Kyempunu	1°39.567′N	31°32.095′E	Farm09	12,309	516	175	1,064
			Farm10	43,986	950	153	1,073
Nyabyeya 2	1°41.265′N	31°33.215′E	Farm11	19,469	642	149	1,101
			Farm12	23,534	878	57	1,092
Panyana	1°41.335′N	31°31.430′E	Farm06	25,826	791	231	1,079
			Farm13	37,018	798	397	1,085

1 At estimated geographic centre of village.

2 Total extent of farm edge adjoining forest.

3 Above mean sea level at estimated geographic centre of farm.

All study farms adjoined forest and were selected for (a) vulnerability to crop-raiding [4], [16], [22], (b) extensive view of forest edges, (c) range and distribution of crops that was representative of local farms, and (d) farmer support for research objectives. The sample of farmers and farms reflected local demographic diversity and variation in farm size [4], [5]; each farmer used guarding as their primary method of crop protection.

**Figure 2:**
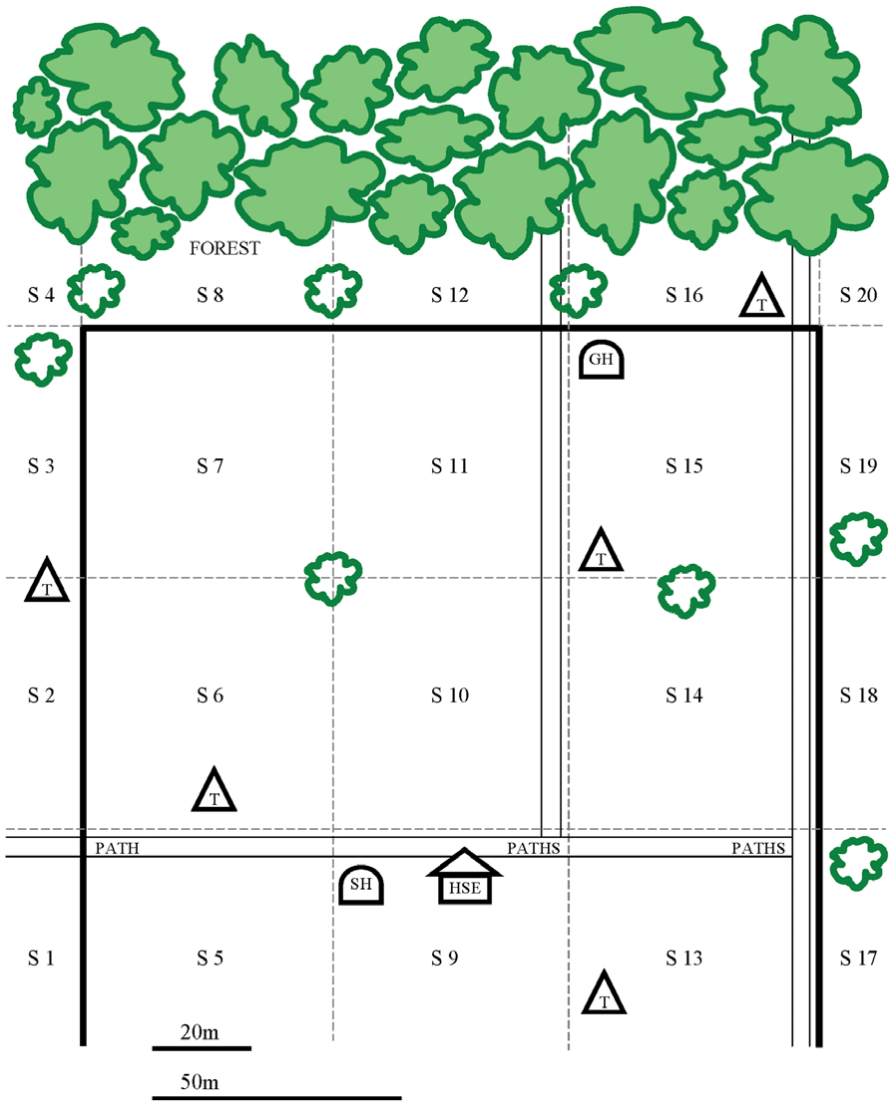
Diagrammatic example of a farm map used by observers. HSE = house. GH = guard hut. SH = storage hut. T = termite mound. S = sector. Solid black lines = farm boundary. Green objects = trees.

Farms were mapped using 30 m measuring tapes, a GPSMAP® 60CS global positioning system unit, and MapSource® 6.11.5 (Garmin Ltd., Olathe, USA). Data compiled were topographic features, structures, perimeter characteristics, edge lengths, field areas, crop distribution, and crop abundance (derived from plot counts). Median farm extent perpendicular to forest was 183 m (range 41 m to 419 m); median length of farm-forest edge was 146 m (range 72 m to 312 m). Distances from farm edges to reference features or structures (e.g. trees, termite mounds, paths, or huts) were recorded to aid distance estimation. Each farm map included numeric sectors to describe locations rapidly and consistently; sector boundaries coincided with features or structures (Figure 2).

Crops were along the farm-forest edge of all study farms and covered a median of 88% of farm area. Maize (*Zea mays*) and beans (*Phaseolus vulgaris*) predominated across study farms (73% of total crop area) and locally; sorghum (*Sorghum bicolor*), bananas (*Musa* spp), and cassava (*Manihot esculenta*, *Manihot palmata*) were also abundant. Median stem density per square metre was 2.9 for maize and 4.9 for beans. Each study farm adjoined other farms with crops, and therefore none were especially vulnerable to raiding due to isolation [6]. Timing of crop planting, growth, and maturity was equivalent across study farms and adjoining farms.

### Data Collection

Data reported here were collected from February to September 2006 by GW and four other observers, including three Ugandans skilled in English and local languages. GW trained and assessed observers to ensure standardised procedures; all observers attained 100% accuracy for crop and primate identification. A crop-raiding event (CRE) was defined as when one or more individuals of a species entered a farm (i.e. crossed a farm boundary), interacted with one or more crop stems, and left the farm. A CRE commenced when the first individual entered the farm and ended when the last individual exited; duration was measured in seconds using digital stop-watches. A stem was one plant, stalk, or fruit of a crop, and a crop was deemed targeted if more-abundant or more-accessible crops were bypassed to acquire it. Primate age categories were adult (full species-sex-specific size), sub-adult (not fully grown, beyond infant development, exhibits independent behaviour frequently), or infant (developmentally small and dependent, carried frequently, maintains close proximity to adults).

Data were collected using all-occurrences continuous sampling [24] and included for each CRE: (1) time and distance to nearest human when the first individual entered the farm, (2) time when each additional individual entered the farm, (3) age-category and sex of each individual, (4) farm entry point(s), (5) incidence and location(s) of crop interaction, including type(s) of crop, (6) time when each individual exited the farm, (7) time and distance to nearest human when the last individual exited the farm, (8) farm exit point(s), (9) total number of individuals entering the farm and total number remaining at the forest edge, (10) maximum distance any individual travelled onto the farm, and (11) median distance that most individuals (i.e. just over 50%) travelled onto the farm. Data regarding the behaviour of farmers and other humans on farms were also collected using all-occurrences continuous sampling. These data included presence or absence of humans on farms, nature of on-farm human activity, extent of guarding behaviour, and responses to crop-raiding primates. Crop damage was determined by counting stems interacted with, consumed, and/or carried by primates during CREs. Usually two observers worked together and rotated data-recording to avoid fatigue. Binoculars were often used to aid observations.

Observations were from hides affording a continuous view of on-farm and forest edge activity while rendering observers inconspicuous to wildlife. As agreed with farmers, observers did not respond to animals entering farms and did not disclose raiding activity to any people on farms. Farmers’ guarding huts were conspicuous and not used for observations because this may appear as guarding, thereby influencing primate behaviour, biasing data, and suggesting that humans in guarding huts do not respond to raiding. *Ad libitum* data indicated that observer presence did not modify wildlife or farmer behaviour [9]. All data were collected in accordance with institutional ethics requirements, established ethical guidelines for social and primate research, and with the consent and support of village councils and participating farmers.

A total of 1,803 hours of observations were conducted over 346 sessions, each 5 to 6 hours in duration. Sampling was representative across farms, months, days of the week, and time of day from sunrise to sunset; schedules at each farm were varied to avoid confounds from predictable sampling patterns. Inter-observer reliability and distance estimates exceeded 95% concordance during bi-monthly assessments; each observer’s estimates were within 10% of measured distances and considered sufficiently accurate for analysis [25].

### Data Analysis

Data were analysed using SPSS 14 for Windows (SPSS Inc., Chicago, USA); tests were two-tailed and results considered statistically significant when *p*≤0.05. Kolmogorov-Smirnov and Schapiro-Wilk tests confirmed non-normal distributions of data, and hence non-parametric tests were used for primary analysis. Median values describe central tendency. For partial correlation and multiple regression analysis, values for continuous variables were logarithmically (base-e) transformed and Q-Q plots confirmed normality after transformation. Regression models were built using forward, backward, stepwise, and direct-entry methods; in each case stepwise models aligned with observed values and were reported. Model results are reported as R^2^ values (proportion of variance accounted for), beta values (contribution to the model), *t* statistics (statistical significance of the contribution), tolerance values (where values <0.2 indicate problems of collinearity), and regression equations (the combination of variables best accounting for observed outcomes) [26]. Regression equations were converted from logarithmic values to describe contributions of CRE parameters to crop damage.

## Results

### Raiding Species

Across all study farms, primates were involved in 96% of observed CREs by wildlife (n = 227) and accounted for 99% of crop stems damaged (n = 4,168). Raiding primate species were olive baboons (*Papio anubis*), red-tailed monkeys (*Cercopithecus ascanius schmidti*), vervet monkeys (*Chlorocebus aethiops*), blue monkeys (*Cercopithecus mitis stuhlmanni*), chimpanzees (*Pan troglodytes schweinfurthii*), and black & white colobus monkeys (*Colobus guereza occidentalis*). Other species observed raiding, accounting for only nine CREs in total, were ground squirrel (*Xerus erythropus*), banded mongoose *(Mungos mungo*), casqued hornbill (*Bycanistes subcylindricus*), common duiker (*Sylvicapra grimmia*), and guineafowl (*Numida meleagris*). Values for raiding parameters varied across primate species (Table 2); the typical primate CRE involved 3 individuals raiding crops from 15 m to 20 m onto a farm for almost 8 minutes.

**Table 2:** Values of parameters for crop-raiding events by each primate species.

		Duration of crop-raidingevent (min:sec)		Median distance travelledonto farm (m)		Maximum distancetravelled onto farm (m)		Number of individuals inraiding group	
Species	n CREs	Median	Range	Median	Range	Median	Range	Median	Range
Baboon	76	8∶27	2∶03–54∶52	25.0	1–110	37.5	1–120	5	1–28
Blue monkey	26	9∶34	0∶35–25∶14	15.0	1–50	18.0	2–70	2	1–6
B&W colobus	6	7∶36	4∶32–18∶37	15.0	15–45	18.0	18–50	2–3	2–8
Chimpanzee	12	7∶33	1∶18–81∶09	45.0	12–100	50.0	12–100	2–3	1–10
Red-tailed monkey	58	8∶18	1∶05–78∶22	12.0	2–55	18.0	2–65	3	1–10
Vervet monkey	40	6∶18	1∶20–53∶35	11.0	2–55	15.0	3–90	2–3	1–8
**All primates**	218	7∶47	0∶35–81∶09	15.0	1–110	20.0	1–120	3	1–28

### Duration of Raid

**Figure 3:**
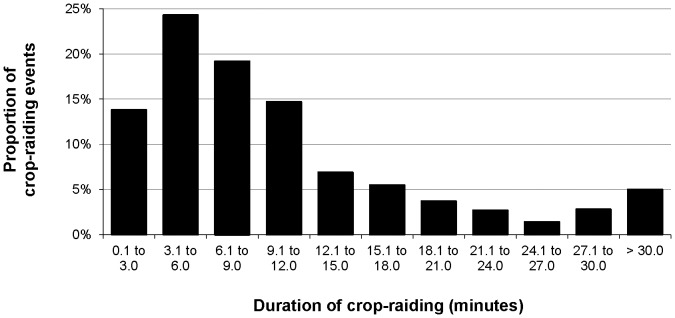
Relative frequency of raid durations across all primate CREs (n = 218).

Median raid duration was 7 minutes 47 seconds and not significantly different between species (Kruskal-Wallis test, χ^2^ = 3.690, *df* = 5, *p* = 0.595). Raids by single individuals were significantly shorter (median 3 minutes 34 seconds) than raids by two or more individuals (median 8 minutes 51 seconds) (Mann-Whitney *U* test, n_(single)_ = 41 n_(two+)_ = 177, *U* = 1671.0, *p*<0.001). Most CREs (58%) were 3 to 12 minutes in duration; almost 80% were shorter than 15 minutes (Figure 3).

### Distance Travelled onto Farm

**Figure 4:**
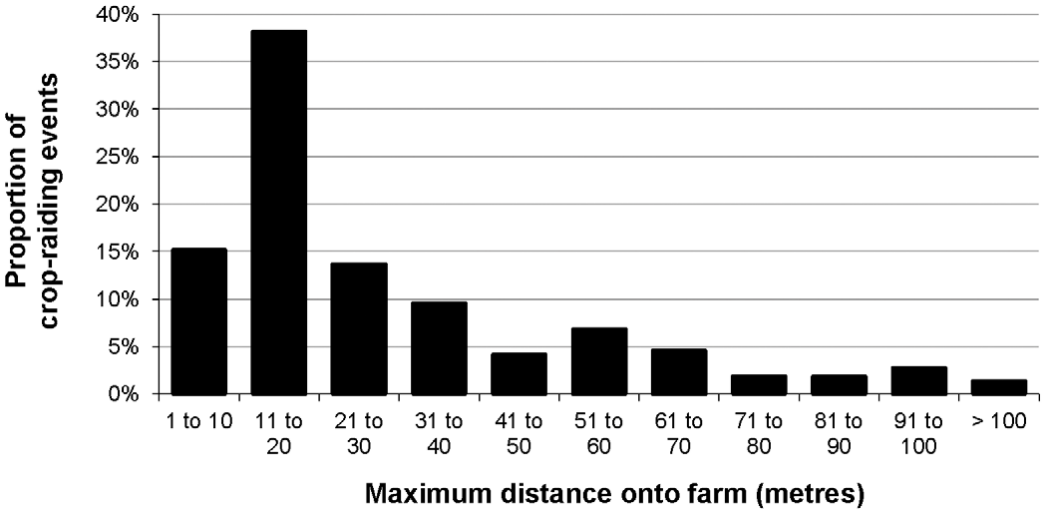
Relative frequency of maximum distances travelled onto farms by any individual in a raiding group across all primate CREs (n = 218).

**Figure 5:**
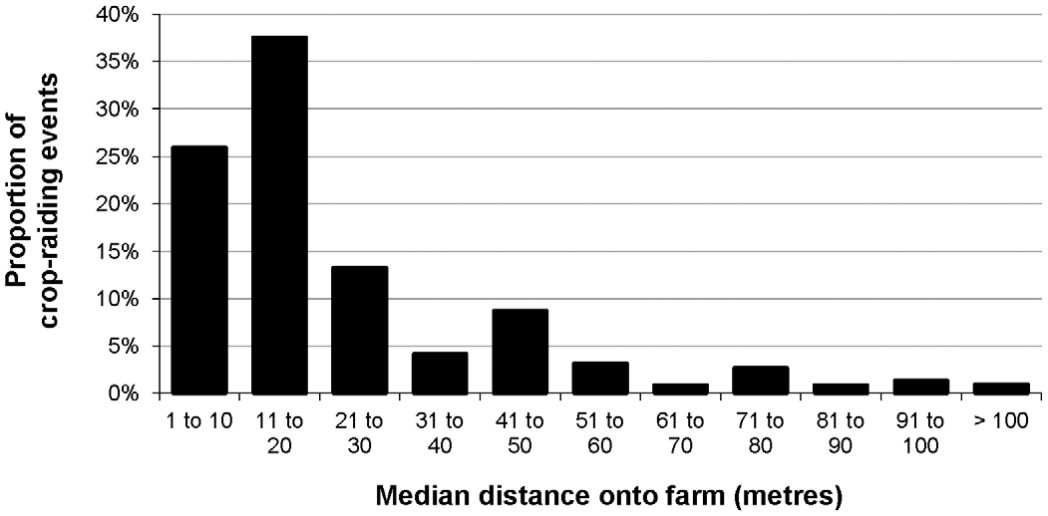
Relative frequency of median distances travelled onto farms by most individuals in a raiding group across all primate CREs (n = 218).

Because study farms adjoined forest, the distances travelled onto farms by raiding primates were also distances travelled from the forest. Maximum on-farm travel distances were 30 m or less during 67% of CREs but exceeded 50 m during 19% of CREs (Figure 4). Median distances were 30 m or less during 77% of CREs but exceeded 50 m during 10% of CREs (Figure 5). Distances aligned directly with relative body size and larger-bodied species (chimpanzees and baboons) travelled furthest (Kruskal-Wallis tests: maximum distance χ^2^ = 49.833, median distance χ^2^ = 49.963, *df* = 5, *p*<0.001). All species raided near farm-forest edges; only baboons and chimpanzees travelled beyond 100 m from forest. Although red-tailed monkeys travelled up to 65 m onto farms, they only ventured more than 30 m from forest in groups and travelled significantly further when multiple individuals raided (Mann-Whitney *U* tests n_(single)_ = 15 n_(two+)_ = 43: maximum distance *U* = 160.5, *p*≤0.004; median distance *U* = 195.0, *p*≤0.023).

### Number of Individuals Raiding

A total of 1,115 primates (not necessarily identified individuals) were counted at forest edges immediately prior to or during CREs. Of these, 939 (84%) entered farms, including all black & white colobus monkeys (n = 23), 96% of chimpanzees (n = 46), 87% of vervet monkeys (n = 112), 84% of baboons (n = 485), 79% of red-tailed monkeys (n = 208), and 70% of blue monkeys (n = 65). Red-tailed monkeys and blue monkeys were significantly more likely than other primates to remain near the forest edge while conspecifics raided (Kruskal-Wallis test, χ^2^ = 50.248, *df* = 5, *p*<0.001). Number of individuals entering a farm correlated positively with number at the forest edge prior to raiding (Spearman’s Rank Correlation Coefficient, *r*_s_ = 0.807, n = 218, *p*<0.001); this was the case when humans were present on the farm (Spearman’s Rank Correlation Coefficient, *r*_s_ = 0.803, n = 163, *p*<0.001) and also when humans were not present (Spearman’s Rank Correlation Coefficient, *r*_s_ = 0.814, n = 55, *p*<0.001).

**Figure 6:**
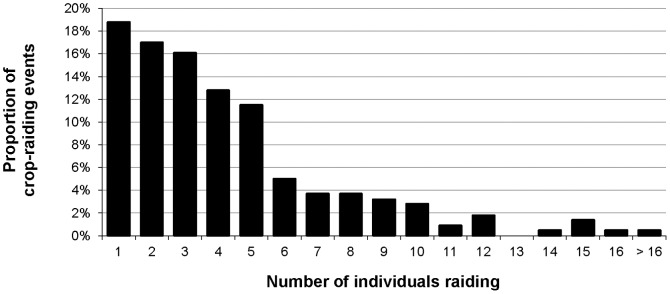
Relative frequency of raiding-group sizes across all primate CREs (n = 218).

Most CREs (52%) involved three or fewer individuals, 36% were by a single individual or pair, and only 23.9% involved more than five individuals (Figure 6). Baboons raided in significantly greater numbers than other species (Kruskal-Wallis test, χ^2^ = 41.914, *df* = 5, *p*<0.001); however, most baboon raiding groups were small compared to maximum sizes and 70% comprised fewer than ten individuals. Blue monkeys, red-tailed monkeys, and vervet monkeys were more likely than other species to raid alone (Kruskal-Wallis test, χ^2^ = 15.785, *df* = 5, *p*≤0.007).

### Influence of Farmer Behaviour

The amount and quality of guarding observed during the study varied between farms and did not prevent raiding of crops. Farmers and/or other humans were present on study farms during 75% (n = 163) of primate CREs. Duration of raid, maximum distance travelled onto farm, median distance travelled onto farm, and number of individuals raiding did not differ significantly according to whether or not humans were present on the farm (Mann-Whitney *U* tests n_(present)_ = 163 n_(absent)_ = 55: duration *U* = 5422.5, *p* = 0.379; maximum distance *U* = 4177.0, *p* = 0.449; median distance *U* = 4422.0, *p* = 0.881; number raiding *U* = 4612.0, *p* = 0.746). Farmers were virtually equally as likely to actively respond to a raid (51% of CREs; n = 111) as to not actively respond (49% of CREs; n = 107). The only observed effect of farmers’ responses on primate raiding behaviour was to stop further travel onto the farm (other than returning to the forest) and terminate the raiding event (thereby determining CRE duration). All instances of farmers responding to raids resulted in raiding primates moving closer to the farm-forest boundary and leaving the farm. Duration of raid did not differ significantly according to whether or not farmers responded (Mann-Whitney *U* test, n_(respond)_ = 111 n_(not respond)_ = 107, *U* = 5111.0, *p* = 0.076). See Wallace [9] for analysis of other aspects of farmer behaviour not impinging upon relationships between primate CRE parameters and amount of crop loss.

### Association between CRE Parameters

Primates allocated most on-farm time to interacting with and eating crops, typically only travelling further onto farms to access more or targeted crops [9]. Therefore, stem damage was expected to increase as duration of raid, distance travelled onto farm, and/or size of raiding group increased. Number of stems damaged correlated positively with size of raiding group (*r*_s_ = 0.819, n = 218, *p*<0.001), duration of raid (*r*_s_ = 0.685, n = 218, *p*<0.001), maximum on-farm travel distance (*r*_s_ = 0.374, n = 218, *p*<0.001), and median on-farm travel distance (*r*_s_ = 0.269, n = 218, *p*<0.001). Partial correlation analysis confirmed each parameter (i.e. raiding group size, raid duration, and on-farm travel distance) interlinked (Table 3). Maximum and median travel distances were highly associated, as expected; other parameters were not. All inter-correlations were positive, indicating that larger raiding groups travelled further onto farms and raided for longer durations compared to small groups or lone raiders. Partial correlation analysis also showed that number of stems damaged only associated significantly with duration of raid and number of individuals raiding (Table 3), suggesting crop loss is not directly related to distance travelled onto farm when other parameters are controlled for.

**Table 3:** Partial correlations between CRE parameters and crop damage. Figures are correlation coefficient and *p*-value; statistically significant results are in bold.

Parameters	Median on-farm distance	Maximum on-farm distance	Number of individuals raiding	Crop Stem damage
**Duration of raid**	**0.183**	**0.236**	**0.250**	**0.541**
	***p*** **≤0.007**	***p*** **<0.001**	***p*** **<0.001**	***p*** **<0.001**
**Median on-farm distance**		**0.975**	**0.390**	0.041
		***p*** **<0.001**	***p*** **<0.001**	*p* = 0.545
**Maximum on-farm distance**			**0.443**	0.027
			***p*** **<0.001**	*p* = 0.693
**Number of individuals raiding**				**0.686**
				***p*** **<0.001**

### Accounting for Crop Damage

Because primate raiding behaviour is often context dependent [9] it is unlikely that CRE parameters contribute equally to crop loss during a raid. Four models accounting for the number of stems damaged by primates were derived using multiple regression: (i) all types of crop, (ii) maize and beans, (iii) maize only, and (iv) beans only (Table 4). In each case loss was predominantly tied to number of individuals raiding and duration of raid; maximum or median on-farm travel distance did not predict stem damage significantly. Each model accounted for a major proportion (74.6% to 87.2%) of total variance in damage, and high tolerance values (0.708 to 0.840) confirmed they were not compromised by collinearity between variables. Regression equations describing parameter contributions to crop loss are:

**Table 4:** Values for each multiple regression model accounting for the number of crop stems damaged by primates during CREs.

Model: all crops	R^2^	B	SE	Beta	*t*	*p*	Tolerance
*Step 1*	0.632						
Constant		1.062	0.085				
Log_e_ individuals raiding		1.167	0.061	0.795	19.267	<0.001	1.000
*Step 2*	0.746						
Constant		–1.716	0.293				
Log_e_ individuals raiding		0.901	0.057	0.614	15.708	<0.001	0.775
Log_e_ duration of raid		0.504	0.051	0.383	9.790	<0.001	0.775
**Model: maize & beans**	**R^2^**	**B**	**SE**	**Beta**	***t***	***p***	**Tolerance**
*Step 1*	0.648						
Constant		1.050	0.089				
Log_e_ individuals raiding		1.177	0.064	0.805	18.264	<0.001	1.000
*Step 2*	0.777						
Constant		–2.042	0.312				
Log_e_ individuals raiding		0.918	0.057	0.628	15.986	<0.001	0.804
Log_e_ duration of raid		0.556	0.055	0.400	10.171	<0.001	0.804
**Model: maize only**	**R^2^**	**B**	**SE**	**Beta**	***t***	***p***	**Tolerance**
*Step 1*	0.697						
Constant		0.907	0.090				
Log_e_ individuals raiding		1.084	0.065	0.835	16.632	<0.001	1.000
*Step 2*	0.869						
Constant		–2.389	0.270				
Log_e_ individuals raiding		0.849	0.047	0.654	18.094	<0.001	0.840
Log_e_ duration of raid		0.582	0.046	0.452	12.514	<0.001	0.840
**Model: beans only**	**R^2^**	**B**	**SE**	**Beta**	***t***	***p***	**Tolerance**
*Step 1*	0.758						
Constant		1.326	0.127				
Log_e_ individuals raiding		1.340	0.092	0.871	14.590	<0.001	1.000
*Step 2*	0.872						
Constant		–1.425	0.368				
Log_e_ individuals raiding		1.006	0.080	0.654	12.571	<0.001	0.708
Log_e_ duration of raid		0.521	0.067	0.401	7.721	<0.001	0.708

#### All crops

Number of stems damaged = –0.723 + (number of individuals raiding × 3.381) + (duration of raid in seconds × 0.010).

#### Maize & beans

Number of stems damaged = –4.158 + (number of individuals raiding × 3.757) + (duration of raid in seconds × 0.013).

#### Maize-only

Number of stems damaged = –0.272 + (number of individuals raiding × 2.180) + (duration of raid in seconds × 0.007).

#### Beans-only

Number of stems damaged = –13.723 + (number of individuals raiding × 7.383) + (duration of raid in seconds × 0.024).

Finer-scale regression models were derived for each frequently-raiding species and the crop they raided most often. Although these species-crop-specific models were exploratory due to small sample sizes [26], key parameters were again number of individuals raiding and duration of raid, accounting for large proportions of variance in damage (Table 5).

**Table 5:** Coefficients of determination (R^2^ values) for species-crop-specific multiple regression models.

Primate species	Crop raided	n CREs	Coefficient of determination
Baboon	Maize	48	0.790
Blue monkey	Maize	23	0.859
Red-tailed monkey	Maize	39	0.945
Vervet monkey	Beans	31	0.913

Stem damage (a) per CRE and (b) per unit of each CRE parameter (i.e. per minute of raiding, per metre onto farm, or per raiding individual) differed between species (Table 6). Damage per CRE was greatest for baboons and black & white colobus monkeys, and least for blue monkeys. Crop loss per unit of each parameter reflected variation in parameter values across species. Baboons, vervet monkeys, and black & white colobus monkeys damaged more stems per unit of each parameter than other species, particularly per raiding individual. Rates of damage per parameter for chimpanzees, blue monkeys, and red-tailed monkeys were comparatively low.

**Table 6:** Crop stem damage per CRE and per unit of each CRE parameter across all primate raids (n = 218).

	Number of crop stems damaged			
Species	Per CRE	Per minute of raiding	Per metre onto farm^1^	Per raiding individual
Baboon	30.5	2.7	0.8	4.8
Blue monkey	9.2	1.0	0.5	3.7
B&W colobus	25.3	2.5	1.3	6.6
Chimpanzee	14.8	0.9	0.3	3.8
Red-tailed monkey	13.9	1.2	0.9	3.9
Vervet monkey	19.1	2.0	1.1	6.8
**All primates**	20.4	1.8	0.8	4.7

1 Median distance travelled onto farm.

### Age Categories of Crop-raiding Primates

Significantly more adults than sub-adults, and more sub-adults than infants, were observed on study farms during CREs (Mann-Whitney *U* tests: n_(sub-adult)_ = 221 n_(adult)_ = 672, *U* = 61159.0, *p*<0.001; n_(infant)_ = 46 n_(sub-adult)_ = 221, *U* = 3286.0, *p*<0.001); this was also the case for each species (chi-square tests, minimum *p*≤0.007) (Table 7). Almost 72% of raiders were adult, including 83% of guenons, and adults were a majority in 92% of CREs by multiple individuals (n = 177). Baboons and chimpanzees raided in mixed age-category groups significantly more frequently than other species (Kruskal-Wallis test, χ^2^ = 28.539, *df* = 5, *p*<0.001), and baboon raiding groups were most diverse (Kruskal-Wallis test, χ^2^ = 53.645, *df* = 5, *p*<0.001) (Table 8). At least one infant was on-farm during 24 baboon raids and one chimpanzee raid; infants were occasionally near forest edges and accompanied by an adult during raids by other primates, but did not enter farms. Almost two-thirds of baboon raiding groups included one or more sub-adults.

**Table 7:** Proportion of the total number of on-farm primates during CREs (n = 939) that were adults, sub-adults, or infants.

	Proportion of total number of individuals on farms (%)		
	Adults	Sub-adults	Infants
Baboon	62.0	28.7	9.3
Blue monkey	86.2	13.8	0.0
B&W colobus	78.3	21.7	0.0
Chimpanzee	73.9	23.9	2.2
Red-tailed monkey	83.2	16.8	0.0
Vervet monkey	80.4	19.6	0.0
**All primates**	71.6	23.5	4.9

**Table 8:** Age-category composition of primate raiding groups during CREs (n = 218).

	Composition of crop-raiding group				
	Single adult only	Adults only^1^	Adults and sub-adults	Adults and infants	Adults, sub-adults, and infants
Species	% CREs	% CREs	% CREs	% CREs	% CREs
Baboon	7.9	28.9	39.5	5.3	26.3
Blue monkey	38.5	76.9	23.1	0.0	0.0
B&W colobus	0.0	66.7	33.3	0.0	0.0
Chimpanzee	16.7	41.7	50.0	0.0	8.3
Red-tailed monkey	25.9	63.8	36.2	0.0	0.0
Vervet monkey	20.0	62.5	37.5	0.0	0.0
**All primates**	18.8	51.8	36.7	1.9	9.6

1 Includes single-adult-only CREs.

All on-farm adult and sub-adult primates damaged at least one crop stem. Although infants interacted with crops intermittently by pulling or biting stems, they usually travelled or rested near an adult female, or engaged in play behaviour with other infants or sub-adults, suggesting they were not anxious during CREs. Females with an infant were particularly vigilant on farms, usually first to return to the forest carrying their infant, and first to flee in response to human actions. Sex of raiding individual was not determined with sufficient reliability for analysis; however, counts of male (n = 62) and female (n = 51) adult baboons on-farm during CREs did not differ significantly (chi-square test, χ^2^ = 1.071, *df* = 1, *p* = 0.301). While significantly more crop stems were damaged by mixed-age groups than by adults-only groups, the former also comprised more individuals, travelled further onto farms, and raided for longer durations (Mann-Whitney *U* tests n_(adults)_ = 73 n_(mixed)_ = 104: stems *U* = 1133.5, *p*<0.001; individuals *U* = 598.0, *p*<0.001; maximum distance *U* = 2877.0, *p*<0.001; median distance *U* = 3079.5, *p*≤0.032; duration *U* = 2354.0, *p*<0.001).

### Multiple versus Single Raids

**Figure 7:**
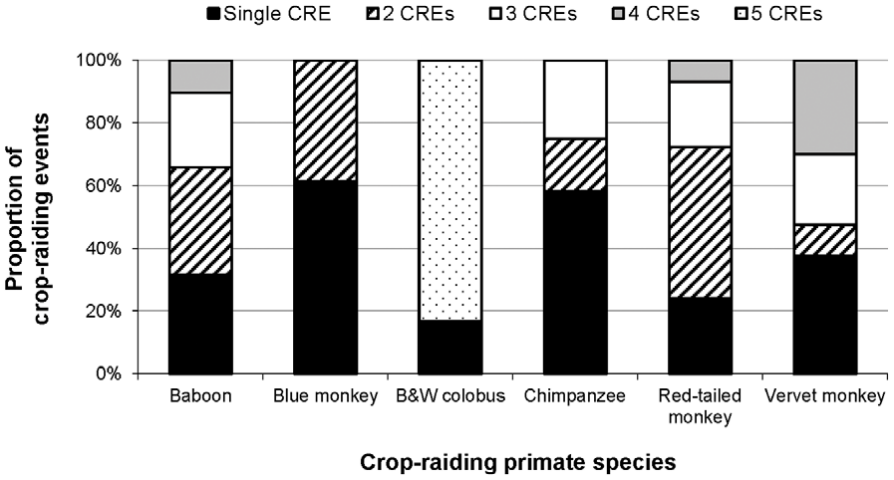
Proportion of CREs by each primate species that were single raids or within a series of multiple-CREs.

A significantly greater proportion of raids (65%; n = 141) were in series rather than single raids (chi-square test, χ^2^ = 18.789, *df* = 1, *p*<0.001); 79% of these were within a 2-CRE or 3-CRE series. Vervet monkeys, red-tailed monkeys, and baboons had diverse multiple-CRE profiles (Figure 7) and raided in series significantly more often than other species (Kruskal-Wallis test, χ^2^ = 27.387, *df* = 5, *p*<0.001). Single raids (n = 77) were most likely to involve one raiding individual (Kruskal-Wallis test, χ^2^ = 12.976, *df* = 5, *p*≤0.024), indicating groups often continue to raid whereas single individuals do not. However, crop damage per CRE did not differ significantly between single raids and raids in series. Raiding in series was not associated with non-detection by farmers, suggesting that farmers’ responses often failed to deter primates from returning.

## Discussion

The prevalence of crop damage by primates across study farms meant that insights about the parameters of primate CREs were integral to understanding the dynamics of raiding. Variability in duration of raid confirmed each species carried out hit-and-run as well as extended raids, as also reported by Maples et al. [27]; Crockett and Wilson [28], Warren [29], Priston [30], and Hockings [31]. Although many CREs were terminated by farmers’ responses, differences in raid duration could reflect adaptation of raiding tactics to perceived on-farm risks, such as probability of detection. Whereas other studies observed that primates predominantly raided crops within 10 m of farm-forest edges [29], [32], [33], median on-farm travel distances during this study exceeded 10 m for each species and were consistent with Naughton-Treves [7]. This suggests that distances travelled onto farms (and hence minimum buffer widths to deter travel) are site specific, particularly because Warren [29] also observed olive baboons.

The positive relationship between primate body size and on-farm travel distance indicates baboons and chimpanzees were more comfortable (or less threatened) away from forest than smaller-bodied species. This might be because baboons and chimpanzees are more terrestrial, or their mass, strength, and average group-size reduces fear of humans, even beyond typical habitat [6], [34], [35], [36]. Primates usually remain near the edges of high-risk habitat [37], [38], suggesting baboons and chimpanzees did not always regard study farms as dangerous places. Similarly, greater travel distances for groups compared to single raiders could have been due to perceived risk because primates typically travel in larger numbers under higher-risk conditions [39], [40], [41]. Planting a crop relatively far from forest is often considered an option to minimise the likelihood of the crop being raided by wildlife [4], [16]. Our data demonstrate species differences in on-farm travel distances for raiding primates. Accordingly, the deterrent value of planting crops in fields relatively far from forest edges probably depends on which primates raid each crop.

The results indicate most primates at forest edges prior to or during CREs were present to participate in raiding. Red-tailed monkeys and blue monkeys were more likely than other species to only observe. Reports of one or two sentinels remaining at the forest edge when baboons raid [6], [14] suggest active involvement in raiding by individuals outside of farms and highly organised, cooperative tactics. However, sentinel behaviour can only be inferred from vigilance and scanning directed over a farm, and was observed rarely during the study (primarily by blue monkeys or red-tailed monkeys and only once by a baboon). Although sentinels were high in trees affording a broad view of on-farm activity, they did not alarm call when farmers approached raiders.

Crop-raiding was not an activity that all members of primate social groups engaged in. Most raids involved small groups relative to species-specific norms for non-raiding activity, and median raiding-group sizes were smaller than typical for primate social groups [42], [43], [44]. Baboon raiding-group sizes aligned with Warren [29], where mean size was 5 (±3) individuals and markedly smaller than social-group size. Although study farmers stated that baboons and red-tailed monkeys usually raid in large groups [4], [6], [29], farmers’ perceptions can differ from observational data due to imperfect detection of raids. Farmers detected relatively large groups most frequently and regularly failed to detect CREs by one to three individuals [9]; raiding in small groups may therefore be effective to avoid detection [15], [27], [45]. For most primates, crop-raiding alone is probably a tactical behaviour to minimise risks while maximising individual returns.

The regression model for all crops raided by primates estimates stem damage generally. It also reflects the crop mix and range of raiding species it is derived for, so that transferability depends on similarity across sites and contexts. While the model incorporates the broad variety of crops raided, it is unlikely to be best fit for specific crops because primates were observed to consume stems of different crops at different rates per unit of time. Compared to the all-crops model, the maize & beans model provides an improved estimate of crop loss during primate CREs because it is attuned to crop prevalence; maize and beans were predominant and raided most frequently by almost all species. The maize & beans model retains broad applicability while accounting for a major proportion of local stem damage.

However, for either crop the maize & beans model is probably skewed towards (a) rates of damage to beans (i.e. many stems consumed per unit of time) for CREs of relatively short duration and (b) rates of damage to maize (i.e. few stems consumed per unit of time) for CREs of extended duration, irrespective of number of individuals raiding. Because the maize-only and beans-only models incorporate crop-specific rates of damage they align more closely with observed maize or beans loss than either other model. This is evident in greater coefficients of determination (R^2^ values) for the maize-only and beans-only models compared to the all-crops and maize & beans models (i.e. 0.869 and 0.872 versus 0.746 and 0.777 respectively).

Precision in accounting for stem damage during CREs improved as regression models became increasingly crop specific. Similarly, models specific to each species that raided frequently and the crop they raided most often also had high coefficients of determination, albeit with smaller sample size. The lower coefficient for the baboon & maize model reflects the greater diversity of crops raided by baboons compared to other species. These results suggest it is possible to derive detailed models to understand and predict context-specific crop loss with sufficient parameter data.

Regression models indicate the relative importance of variables for explaining outcomes but do not establish causation [46]. Although the key parameters determining crop loss were raiding-group size and CRE duration, exclusion of on-farm travel distances from models does not mean these variables failed to impact stem damage entirely; rather, their influence was probably secondary. Travelling progressively further from forest was often necessary to access more stems during raids. Distances were tied to crop location and preference when baboons or chimpanzees targeted mangos, papaya, or jackfruit, usually grown relatively far from forest. Similarly, variables determining whether and how quickly farmers detect CREs could impact crop loss by influencing raid duration.

As expected from between-species variation in parameter values, stem damage per CRE as well as per unit of each parameter (i.e. per minute of raiding, per metre onto farm, or per raiding individual) was relatively species-specific. In particular, baboons, vervet monkeys, and black & white colobus monkeys often damaged crops quickly and extensively. Rates of damage also reflect interactions between parameters; for example, baboons were likely to cause more crop loss than other primates per CRE and per minute of raiding because they typically raided in greater numbers. This level of analysis discloses species and/or contextual differences in raiding behaviour, including how damage may vary with changes in parameter values due to modified raiding tactics, perhaps in response to deterrent interventions.

Crop-raiding was an adult-led and adult-oriented activity for each species. However, adult predominance does not characterise social-group composition for these primates [42], [43], further confirming that not all group members raided crops. Only baboon raiding groups regularly comprised individuals across all age categories. Absence of infants when most primates, and all guenons, raided might reflect species-specific tactics and raiding-group size, perceived on-farm dangers, and/or age-related differences in diet.

Although recent studies also report adult primates raiding and leading CREs most frequently [29], [30], [32], [47], early research identified sub-adults as main raiders [15], [45], [48], [49]. While raiding by sub-adults could be driven by comparatively high rates of exploratory behaviour or risk-taking [50], [51], this was rare and observed only for baboons and chimpanzees. However, perceptions of risk may influence the age composition of primate raiding groups; for example, adult females with infants consistently raid least frequently, possibly because they are more cautious [31], [52]. This was the case for all species except baboons, indicating baboons restrict the composition and size of raiding groups less than other primates. Absence of infants and hence adult females with infants during most CREs also suggests more males than females raided. Elephants (*Loxodonta africana*, *Elephas maximus*) and wild boars (*Sus scrofa*) exhibit similar behaviour [53], [54], [55], which could characterise many raiding species.

Presence and active raiding by adults and sub-adults during many CREs suggests the skills and tactics of crop-raiding are transferred through imitation and social learning [56], [57], as reported for elephants [58]. Because crops provide greater nutrition than many natural primate foods, consuming crops might also allow sub-adults to grow more quickly than normal and benefit from larger body size [15], [16]. The diverse composition of baboon raiding groups, on-farm presence of infants, and high rates of raiding by baboons [9] suggests baboons were more comfortable on farms than other primates. Hence, baboons in the study area might learn to raid earlier in development, making them more adept, adaptable, and persistent raiders over time. When primates consume crops regularly, by choice or necessity, they may develop a raiding tradition or culture [59], [60]. The group’s cumulative experience would then manifest as crop-raiding behaviour adapted and finely tuned to local conditions, including farmer behaviour. Primates with extensive raiding history can therefore habituate quickly to crop-protection techniques. Deterrents might require cycling or modification over time to be effective, and farmers may need to monitor raiding to plan their responses.

Although the age-category composition of raiding groups influenced crop loss, the effect was secondary because it interlinked with raiding-group size. Similarly, crop-raiding experience may have influenced CRE duration (perhaps by enabling group members to avoid or delay detection by farmers) and/or rate of damage (possibly through greater efficiency when processing stems). Broad multiple-CRE profiles for vervet monkeys, red-tailed monkeys, and baboons indicate these species raid persistently when opportunities arise. However, variation in damage per raid probably only reflects raiding in series *per se* to the extent that raiders become satiated over consecutive raids. Preferences for raiding in series are probably explained by the energetic efficiency of crop consumption [48], [61], whereby reduced foraging and feeding time allows more time for resting and social behaviour [62], [63]. This provides incentives to raid repeatedly, increasing crop loss.

Demonstration that crop damage by primates is mainly determined by number of individuals raiding and CRE duration has implications for crop protection. Farmers will benefit most from deterrent techniques that discourage raiding by multiple individuals, reduce the size of raiding groups, or decrease the amount of time that primates spend on farms. This involves increasing perceived risks for raiders; for example, by improving farmer detection of raids, impeding or restricting farm entry and exit, increasing the efficacy of farmers’ responses, and/or requiring raiders to be more vigilant on farms.

Furthermore, values for parameters of CREs vary between species, probably reflecting unique raiding tactics according to perceived circumstances. Key CRE parameters can therefore be used as quantifiable yardsticks for assessing the behavioural impact of techniques to deter raiding. Specifically, if primates raid in groups of fewer individuals or for shorter durations at a farm (compared to baseline values) after deterrent introduction, it can be concluded that the deterrent is effective because crop loss per raiding event will be reduced. Efficacy may also be indicated if primates raid over reduced distances at the farm, the age composition of raiding groups is relatively homogenous, or primates rarely raid in series. Assessing CRE parameters provides valid indices of how comfortable primates are on a farm, and is informative for managing and mitigating human-wildlife conflict. The process also confirms the importance of understanding crop-raiding thoroughly in order to address it.

## References

[1] Sitati NW, Walpole MJ (2006) Assessing farm-based measures for mitigating human-elephant conflict in Transmara District, Kenya. Oryx 40: 279–286.

[2] Graham MD, Ochieng T (2008) Uptake and performance of farm-based measures for reducing crop raiding by elephants *Loxodonta africana* among smallholder farms in Laikipia District, Kenya. Oryx 42: 76–82.

[3] Strum SC (2010) The development of primate raiding: Implications for management and conservation. International Journal of Primatology 31: 133–156. 20174437 10.1007/s10764-009-9387-5 PMC2819593

[4] Webber AD (2006) Primate crop raiding in Uganda: Actual and perceived risks around Budongo Forest Reserve [PhD Thesis]. Oxford: Oxford Brookes University.

[5] Hill CM (2005) People, crops, and primates: A conflict of interests. In: Paterson JD, Wallis J, editors. Commensalism and conflict: The human-primate interface. Norman, Oklahoma: American Society of Primatologists. 40–59.

[6] Hill CM (2000) Conflict of interest between people and baboons: Crop raiding in Uganda. International Journal of Primatology 21: 299–315.

[7] Naughton-Treves L (1997) Farming the forest edge: Vulnerable places and people around Kibale National Park, Uganda. Geographical Review 87: 27–46.

[8] Osborn FV, Parker GE (2003) Towards an integrated approach for reducing the conflict between elephants and people: A review of current research. Oryx 37: 80–84.

[9] Wallace GE (2010) Monkeys in maize: Primate crop-raiding behaviour and developing on-farm techniques to mitigate human-wildlife conflict [PhD Thesis]. Oxford: Oxford Brookes University. 528 p.

[10] Hill CM, Osborn FV, Plumptre AJ (2002) Human-wildlife conflict: Identifying the problem and possible solutions. Albertine Rift Technical Report Series No 1 Wildlife Conservation Society.

[11] CARE ITFC, CDC WCS (2003) Reducing the costs of conservation to frontline communities in southwest Uganda. Knowledge Base Review Report. CARE International in Uganda, Institute of Tropical Forest Conservation, Conservation Development Centre, and Wildlife Conservation Society. 130 p.

[12] Paterson JD, Wallis J, editors (2005) Commensalism and conflict: The human-primate interface. Norman, Oklahoma: American Society of Primatologists. 483 p.

[13] Woodroffe R, Thirgood S, Rabinowitz A, editors (2005) People and wildlife: Conflict or coexistence? Cambridge: Cambridge University Press. 497 p.

[14] Maples WR (1969) Adaptive behavior of baboons. American Journal of Physical Anthropology 31: 107–109.

[15] Strum SC (1994) Prospects for management of primate pests. Revue d'Ecologie (La Terre Et La Vie) 49: 295–306.

[16] Naughton-Treves L (1998) Predicting patterns of crop damage by wildlife around Kibale National Park, Uganda. Conservation Biology 12: 156–168.

[17] Saj TL, Sicotte P, Paterson JD (2001) The conflict between vervet monkeys and farmers at the forest edge in Entebbe, Uganda. African Journal of Ecology 39: 195–199.

[18] Warren Y, Buba B, Ross C (2007) Patterns of crop-raiding by wild and domestic animals near Gashaka Gumti National Park, Nigeria. International Journal of Pest Management 53: 207–216.

[19] Conover M (2002) Resolving human-wildlife conflicts: The science of wildlife damage management. Boca Raton: Lewis Publishers. 418 p.

[20] Eggeling WJ (1947) Observations on the ecology of the Budongo rain forest, Uganda. Journal of Ecology 34: 20–87.

[21] Plumptre AJ (1996) Changes following 60 years of selective timber harvesting in the Budongo Forest Reserve, Uganda. Forest Ecology and Management 89: 101–113.

[22] Hill CM (1997) Crop-raiding by wild vertebrates: The farmer's perspective in an agricultural community in western Uganda. International Journal of Pest Management 43: 77–84.

[23] Tweheyo M, Hill CM, Obua J (2005) Patterns of crop raiding by primates around the Budongo Forest Reserve, Uganda. Wildlife Biology 11: 237–247.

[24] Altmann J (1974) Observational study of behavior: Sampling methods. Behaviour 49: 227–267. 4597405 10.1163/156853974x00534

[25] Lehner PN (1996) Handbook of ethological methods. Cambridge: Cambridge University Press. 672 p.

[26] Sokal RR, Rohlf FJ (1995) Biometry: The principles and practice of statistics in biological research. New York: W.H. Freeman. 887 p.

[27] Maples WR, Maples MK, Greenhood WF, Walek ML (1976) Adaptations of crop-raiding baboons in Kenya. American Journal of Physical Anthropology 45: 309–315.

[28] Crockett CM, Wilson WL (1980) The ecological separation of *Macaca nemestrina* and *M. fascicularis* in Sumatra. In: Lindburg DG, editor. The macaques: Studies in ecology, behavior and evolution. New York: Van Nostrand Reinhold. 148–181.

[29] Warren Y (2003) Olive baboons (*Papio cynocephalus anubis*): Behaviour, ecology and human conflict in Gashaka Gumti National Park, Nigeria [PhD Thesis]. Roehampton: University of Surrey. 308 p.

[30] Priston NEC (2005) Crop-raiding by *Macaca ochreata brunnescens* in Sulawesi: Reality, perceptions and outcomes for conservation [PhD Thesis]. Cambridge: University of Cambridge.

[31] Hockings KJ (2007) Human-chimpanzee coexistence at Bossou, the Republic of Guinea: A chimpanzee perspective [PhD Thesis]. Stirling: University of Stirling.

[32] Hansen LK (2003) Influence of forest-farm boundaries and human activity on raiding by the Buton macaque (*Macaca ochreata brunnescens*) [MSc Dissertation]. Oxford: Oxford Brookes University. 46 p.

[33] Priston NEC, Wyper RM, Lee PC (2012) Buton macaques (*Macaca ochreata brunnescens*): Crops, conflict, and behavior on farms. American Journal of Primatology 74: 29–36. 22025206 10.1002/ajp.21003

[34] Wolfheim JH (1983) Primates of the world: Distribution, abundance, and conservation. Seattle: University of Washington Press. 831 p.

[35] Nowak RM (1999) Walker's primates of the world. Baltimore: The Johns Hopkins University Press. 224 p.

[36] Reynolds V (2005) The chimpanzees of the Budongo Forest: Ecology, behaviour, and conservation. Oxford: Oxford University Press. 297 p.

[37] Cowlishaw G (1997) Refuge use and predation risk in a desert baboon population. Animal Behaviour 54: 241–253. 9268454 10.1006/anbe.1996.0466

[38] Hill RA (1999) Ecological and demographic determinants of time budgets in baboons: Implications for cross-populational models of baboon socioecology [PhD Thesis]. Liverpool: University of Liverpool. 282 p.

[39] Miller LE, Treves A (2007) Predation on primates: Past studies, current challenges, and directions for the future. In: Campbell CJ, Fuentes A, Mackinnon KC, Panger M, Bearder SK, editors. Primates in perspective. Oxford: Oxford University Press. 525–543.

[40] Stanford CB (2002) Avoiding predators: Expectations and evidence in primate antipredator behavior. International Journal of Primatology 23: 741–757.

[41] Hill RA, Lee PC (1998) Predation risk as an influence on group size in cercopithecoid primates: Implications for social structure. Journal of Zoology 245: 447–456.

[42] Campbell CJ, Fuentes A, Mackinnon KC, Panger M, Bearder SK, editors (2007) Primates in perspective. Oxford: Oxford University Press. 720 p.

[43] Smuts BB, Cheney DL, Seyfarth RM, Wrangham RW, Struhsaker TT, editors (1987) Primate societies. Chicago: The University of Chicago Press. 578 p.

[44] Rowe N (1996) The pictorial guide to the living primates. New York: Pogonias Press. 263 p.

[45] Forthman Quick DL (1986) Activity budgets and the consumption of human food in two troops of baboons, *Papio anubis*, at Gilgil, Kenya. In: Else JG, Lee PC, editors. Primate ecology and conservation. Cambridge: Cambridge University Press. 221–228.

[46] Foster J, Barkus E, Yavorsky C (2006) Understanding and using advanced statistics. London: Sage Publications. 178 p.

[47] Hockings KJ (2009) Living at the interface: Human-chimpanzee competition, coexistence and conflict in Africa. Interaction Studies 10: 183–205.

[48] Oyaro HO, Strum SC (1984) Shifts in foraging strategies as a response to the presence of agriculture in a troop of wild baboons at Gilgil, Kenya. International Journal of Primatology 5: 371–381.

[49] Saj T, Sicotte P, Paterson JD (1999) Influence of human food consumption on the time budget of vervets. International Journal of Primatology 20: 977–994.

[50] Fairbanks LA (1993) Risk-taking by juvenile vervet monkeys. Behavior 124: 57–72.

[51] Janson CH, van Schaik CP (1993) Ecological risk aversion in juvenile primates: Slow and steady wins the race. In: Pereira ME, Fairbanks LA, editors. Juvenile primates: Life history, development and behavior. New York: Oxford University Press. 57–74.

[52] Fairbanks LA, McGuire MT (1993) Maternal protectiveness and response to the unfamiliar in vervet monkeys. American Journal of Primatology 30: 119–129. 10.1002/ajp.1350300204 31937015

[53] Sukumar R, Gadgil M (1988) Male-female differences in foraging on crops by Asian elephants. Animal Behaviour 36: 1233–1235.

[54] Boitani L, Mattei L, Nonis D, Corsi F (1994) Spatial and activity patterns of wild boars in Tuscany, Italy. Journal of Mammalogy 75: 600–612.

[55] Hoare RE (1995) Options for the control of elephants in conflict with people. Pachyderm 19: 54–63.

[56] Whiten A (2000) Primate culture and social learning. Cognitive Science 24: 477–508.

[57] Nishida T (1987) Local traditions and cultural transmission. In: Smuts BB, Cheney DL, Seyfarth RM, Wrangham RW, Struhsaker TT, editors. Primate societies. Chicago: The University of Chicago Press. 462–474.

[58] Chiyo PI, Cochrane EP (2005) Population structure and behaviour of crop-raiding elephants in Kibale National Park, Uganda. African Journal of Ecology 43: 233–241.

[59] Sapolsky RM, Share LJ (2004) A pacific culture among wild baboons: Its emergence and transmission. PLoS Biology 2: 534–541. 10.1371/journal.pbio.0020106 PMC387274 15094808

[60] Galef Jr BG (2004) Approaches to the study of traditional behaviors of free-living animals. Learning & Behavior 32: 53–61. 15161140 10.3758/bf03196006

[61] Chiyo PI, Cochrane EP, Naughton L, Basuta GI (2005) Temporal patterns of crop raiding by elephants: A response to changes in forage quality or crop availability? African Journal of Ecology 43: 48–55.

[62] Lee PC, Brennan EJ, Else JG, Altmann J (1986) Ecology and behaviour of vervet monkeys in a tourist lodge habitat. In: Else JG, Lee PC, editors. Primate ecology and conservation. Cambridge: Cambridge University Press. 229–235.

[63] Altmann J, Muruthi P (1988) Differences in daily life between semiprovisioned and wild-feeding baboons. American Journal of Primatology 15: 213–221. 10.1002/ajp.1350150304 31968889

